# Evaluation of garlic skin as a forage source for goats: effects on performance, antioxidant capacity, immune function and ruminal health

**DOI:** 10.5713/ab.25.0169

**Published:** 2025-07-11

**Authors:** Xinhong Zhou, Xiaoyun Shen

**Affiliations:** 1 College of Life Science and Agri-forestry, Southwest University of Science and Technology, Mianyang, China; 2 Rural Revitalization Project Center, Guizhou Department of Agriculture and Rural Affairs, Guiyang, China

**Keywords:** Garlic Skin, Goat, Immunity, Metabolites, Microbiome, Rumen Fermentation

## Abstract

**Objective:**

This study aimed to evaluate the effects of garlic skin as a feed ingredient on growth performance, antioxidant capacity, immune function, and rumen health in goats.

**Methods:**

Twelve male black goats with similar body conditions were randomly assigned to two groups. The control group (CON) was fed a basal diet, while the experimental group (GAS) received a diet supplemented with garlic skin for 60 days.

**Results:**

Results showed that goats’ final weight and average daily gain significantly increased in the GAS group compared to the CON group (p<0.05). The GAS group exhibited enhanced activities of total superoxide dismutase, glutathione peroxidase, catalase, and higher total antioxidant capacity levels, while malondialdehyde content significantly decreased (p<0.05). Immunoglobulin A and immunoglobulin G levels were significantly elevated, along with increased concentrations of anti-inflammatory cytokines interleukin-4 and interleukin-10, whereas pro-inflammatory cytokines interleukin-1β, interleukin-6, and tumor necrosis factor-α were significantly reduced (p<0.05). The rumen fluid of GAS group showed significant increases in ammonia nitrogen, acetate, propionate, and total volatile fatty acids, with a reduction in the acetate-to-propionate ratio (p<0.05). Significant improvements were also observed in rumen papilla height, width, and density (p<0.05). 16S rDNA analysis revealed enhanced microbial diversity and enrichment of functional bacterial groups, such as *Firmicutes* and *Christensenellaceae_R-7_group*, involved in fiber degradation and volatile fatty acid production. Key metabolites, including 5-methylthioribose and glucose 6-phosphate, were positively correlated with growth and antioxidant capacity.

**Conclusion:**

In conclusion, garlic skin supplementation enhanced antioxidant and immune function, optimized rumen fermentation, improved microbial composition, and promoted goat health and productivity.

## INTRODUCTION

Garlic (*Allium sativum*) is a functional food and medicinal product widely used around the world. As a medicine, it has properties such as promoting digestion, relieving food stagnation, and exhibiting antibacterial and antiparasitic effects. As a food ingredient, garlic is commonly used as a condiment or for seasoning. Major garlic-producing countries include China, India, Korea, Egypt, and the United States, with a global annual production of approximately 27 million tons [1]. Garlic is rich in bioactive compounds, such as allicin, alliin, and allyl sulfides, which exhibit various beneficial effects, including antibacterial, anti-inflammatory, and immunomodulatory properties; enhance immune cell activity; increase antioxidant capacity; and prevent cancer. Numerous studies have demonstrated that dietary supplementation with garlic products can promote the growth and health of monogastric animals, yielding positive outcomes. For ruminants, one study revealed that supplementing 2.0 g of allicin per day per sheep reduced the population of methanogens and protozoans while improving the abundance of *Fibrobacter succinogenes*, *Butyrivibrio fibrisolvens* and *Ruminococcus flavefaciens*. This dietary intervention reduced methane emissions by 5.95% and increased the total volatile fatty acids (TVFA) content in the rumen [2]. The oral administration of a 2 mL mixture of garlic oil and thyme oil (1:1) daily to Damascus goats resulted in a 10% increase in final body weight (FW). Additionally, these goats presented improved immunity, antioxidant capacity, and overall health compared with the control group, with malondialdehyde (MDA) levels reduced by 50% [3]. Allicin supplementation can improve goats’ antioxidant capacity, change the composition of the rumen microbiota, lessen the harm that high–grain diets cause to the rumen epithelial cell junctions, and shield the goats’ rumen epithelium [4]. Compared with monensin, allicin has been shown to increase the growth performance of goats and has superior effects on increasing their antioxidant capacity and immune function [5]. In summary, garlic products offer various biological benefits for ruminants. Garlic and its components can modulate rumen fermentation parameters, improve nutrient digestibility, reduce rumen protozoa populations, lower methane emissions, and ultimately promote the health and growth of ruminants.

Approximately 25%–30% of garlic byproducts consist of garlic skin and stalks. Among every kilogram of garlic bulb, approximately 760 grams are garlic cloves, while the inner and outer peels account for approximately 240 grams [6]. Thus, garlic byproducts have significant potential as high-quality bioresources. In recent years, feed resource shortages have become increasingly prominent, and the competition for grains between humans and livestock has intensified. Agricultural byproducts are often used as roughage for ruminants to partially replace conventional roughage, offering a cost-effective and efficiency-enhancing solution for livestock production. Garlic skin, a byproduct of garlic processing, contains certain active compounds similar to those in garlic. It is also rich in crude protein and crude fiber, making it an excellent source of dietary fiber. Currently, the use of garlic skin as an economical roughage resource in the livestock industry is gradually increasing. Research has identified active compounds in garlic skin, such as N-trans-feruloyloctopamine, N-trans-coumaroyloctopamine, trans-coumaric acid, guaiacylglycerol-β-ferulic acid ether, and transferulic acid, which exhibit strong DPPH radical-scavenging capacity and significant antioxidant activity [7].

The rumen hosts diverse microbial communities that can directly or indirectly influence the host’s performance, health, and immune status. During the digestion process, the rumen microbiota breaks down dietary fiber into volatile fatty acids (VFAs) that the host absorbs and utilizes. Previous studies have demonstrated that feeding diets containing 8% garlic skin to fattening lambs can improve their growth performance and health status by improving their serum antioxidant capacity and modulating their rumen microbiota composition [8]. Similarly, supplementation with garlic skin increased the relative abundance of *Prevotella*, *Bulleidia*, *Howardella*, and *Methanosphaera* at the genus level in the rumen fluid of fattening lambs while reducing the abundance of *Fretibacterium*. These changes in the rumen microbiome promoted rumen fermentation, thereby increasing the growth performance of the lambs [9].

With the livestock industry developing at a rapid pace, feed resource shortages, and the reduction in and ban of antibiotic use, agricultural byproducts have emerged as potential feed resources and important research focuses in modern animal husbandry. Therefore, we evaluated the effects of garlic skin on goat growth performance, antioxidant capacity, and rumen fermentation. Specifically, we aimed to elucidate how garlic skin supplementation promotes goat rumen health and growth through the rumen microbiota and its metabolites. Additionally, we assessed the impact of garlic skin on rumen fermentation, its feeding value, and its potential for resource utilization.

## MATERIALS AND METHODS

### Animals, diets, and experimental design

A total of 12 healthy male Yudong black goats, aged 3 months with an initial body weight (IW) of 15.37±0.58 kg, were randomly assigned to two groups (n = 6 per group). The goats were randomly distributed into 12 pens, with one goat per pen. The control group (CON) was fed a diet without garlic skin, whereas the experimental group (GAS) was fed a diet containing 16% garlic skin. The feeding trial lasted for 67 days, with the first 10 days designated the adaptation period. The goats were fed the experimental diets twice daily at 08:00 and 17:00, and all the goats had free access to water and light. All the diets were formulated according to the Nutrient Requirements of Meat-type Sheep and Goats (NYT 816--2021), an agricultural industry standard of the People’s Republic of China. The diets and garlic skin formulations and nutritional compositions of both groups are presented in Supplements 1, 2.

### Sample collection

At the end of the trial, blood samples were collected from the jugular vein of all the goats in each group before the morning feeding. After standing, the blood was centrifuged at 3,000 r/min for 10 minutes at 4°C, and the serum was stored at −20°C. At the same time, 100 milliliters of rumen fluid were collected using an oral rumen fluid collector. Fifty millilitres of the rumen fluid were immediately used to measure the pH, while the remaining 50 mL was filtered through four layers of sterile gauze, transferred into a sterile 50 mL polypropylene centrifuge tube and rapidly frozen in liquid nitrogen. The samples were subsequently stored at −80°C for future analysis. Furthermore, approximately 2 cm^3^ of rumen tissue samples were collected from the ventral sac of the rumen, near the papilla-rich area, and preserved in 4% paraformaldehyde solution for further rumen morphological examination.

### Measurement and methods

#### Growth performance

Before the official trial period began, the IW of the experimental goats was recorded. The FW was recorded the morning after the trial period ended. The average daily gain (ADG) was calculated. The daily dry matter intake (DMI) was accurately recorded for each pen, and the amount of dry matter fed and remaining was noted. The DMI per goat was then calculated per pen.

#### Serum biochemical indicators

Using kits from Nanjing Jiancheng Bioengineering Institute and an enzyme-labeled device (Thermo Fisher Scientific), the serum total antioxidant capacity (T-AOC), superoxide dismutase (T-SOD) activity, glutathione peroxidase (GSH-Px) activity, catalase (CAT) activity, and MDA concentration were measured. Commercial ELISA kits (Nanjing Jiancheng Bioengineering Institute) and an enzyme-labeled device (Thermo Fisher Scientific) were used to measure the levels of immunoglobulin G (IgG), immunoglobulin M (IgM), immunoglobulin A (IgA), interleukin-4 (IL-4), interleukin-10 (IL-10), interleukin 1β (IL-1β), interleukin-6 (IL-6), and tumor necrosis factor-α (TNF-α).

#### Rumen fermentation parameters

A portable pH meter was used to measure the rumen contents’ pH directly. Colorimetry was used to measure the concentration of ammonia nitrogen (NH3-N). Gas chromatography was used to determine the amount of VFAs. In short, a sample was vortexed for two minutes after being combined with two milliliters of water (1:3 phosphoric acid solution). Following the addition of 2 mL of ether for extraction, the tube was centrifuged for 20 minutes at 1,500×g. After centrifugation, the ether phase was collected and re-extracted with 2 mL of ether for 10 minutes, followed by centrifugation again. The ether phase was collected after the second extraction, and the two ether extracts were combined, evaporated, and diluted to a final volume of 4 mL for analysis.

#### Rumen morphology measurement

The tissue samples fixed in 4% paraformaldehyde were subsequently trimmed, dehydrated, embedded in paraffin, sectioned, stained with hematoxylin and eosin (H&E), and mounted to obtain high-quality slides for microscopic examination [10]. Target regions of the tissue were imaged at 20× magnification using an Eclipse Ci-L microscope (Nikon), ensuring that the tissue filled the entire field of view and maintaining consistent background lighting across all images. The images were analyzed using Image-Pro Plus 6.0 software (Media Cybernetics) to calculate papilla height, papilla width, and papilla density, with all measurements standardized in millimeters.

#### Rumen microbial 16S rRNA analysis

Rumen fluid samples were immediately snap-frozen and stored at −80°C until analysis. Bacterial DNA was extracted from the rumen fluid using a DNeasy PowerSoil kit (Qiagen) according to the manufacturer’s protocol. DNA concentration and purity were assessed with a NanoDrop 2000 spectrophotometer (Thermo Fisher Scientific), and DNA integrity was confirmed by agarose gel electrophoresis. The V3–V4 hypervariable regions of the bacterial 16S rRNA gene were amplified in a 25 μL PCR reaction using universal primers 343F (5′-TACGGRAGGCAGCAG-3′) and 798R (5′-AGGGTATCTAATCCT-3′). The reverse primer included a unique sample barcode, and both primers were appended with Illumina sequencing adapters. PCR amplicon quality was verified by gel electrophoresis. PCR products were purified using Agencourt AMPure XP beads (Beckman Coulter) and quantified with a Qubit dsDNA Assay Kit (Thermo Fisher Scientific). Concentrations were normalized for sequencing. Sequencing was performed on an Illumina NovaSeq 6000 platform (Illumina) with paired-end 250 bp cycles (OE Biotech). Raw sequencing data were obtained in FASTQ format. Paired-end reads were first demultiplexed and adapter-trimmed using cutadapt. After trimming, reads were filtered to remove low-quality sequences, denoised, merged, and chimeric sequences were identified and removed using DADA2 within QIIME 2. This process generated representative sequences (amplicon sequence variants, ASVs) and an ASV abundance table. Each representative sequence was taxonomically classified by aligning against the SILVA database (ver. 138). Alpha diversity was estimated using the Chao1 index and the Shannon diversity index. Unweighted UniFrac distances were calculated in QIIME 2, and principal coordinate analysis (PCoA) was performed to visualize phylogenetic differences among samples. A phylogenetic tree was also constructed in QIIME 2 based on the UniFrac distance matrix. All 16S rRNA gene amplicon sequencing and downstream analyses were conducted by OE Biotech.

#### Rumen microbial untargeted metabolomics analysis

The samples stored at −80°C were thawed in an ice-water mixture, and 200 μL of each sample was transferred to a 1.5 mL EP tube. A total of 600 μL of methanol–acetonitrile solution (V:V = 2:1, containing mixed internal standards at 4 μg/mL) was added, followed by vortexing for 1 minute. After ten minutes of ultrasonic extraction in an ice-water bath, the samples were left to stand at −40°C for the whole night. Following a 20-minute centrifugation at 12,000×g and 4°C, 150 μL of the supernatant was moved to an LC-MS injection vial for examination. Equal amounts of extraction fluid from each sample were combined to create quality control (QC) samples. A Waters ACQUITY UPLC I-Class plus/Thermo QE HF system was used for the detection. Progenesis QI v3.0 software (Nonlinear Dynamics) was used to treat the raw data. This included normalization, peak alignment, integration, baseline filtering, peak identification, and retention time correction. Principal component analysis (PCA), orthogonal partial least squares discriminant analysis (OPLS-DA), and pathway enrichment analysis were all performed using MetaboAnalyst 5.0 software. Differential metabolites with VIP>1.0, FC>2.0, and p<0.05 were determined to be significant metabolites between the two treatments based on the findings of the OPLS-DA analysis. To find important differential metabolites, Cluster 3.0 software was used to conduct hierarchical clustering analysis (HCA). The Kyoto Encyclopedia of Genes and Genomes (KEGG) database (URL: http://www.genome.jp/kegg/), the Human Metabolome Database (HMDB) online version (URL: https://hmdb.ca), and MetaboAnalyst 5.0 (URL: https://www.metaboanalyst.ca/) were accessed in order to plot enrichment pathway maps for various metabolites [11,12].

### Data analysis

The means±standard deviations represent the experimental outcomes. The independent samples t test in SPSS 23.0 software was used to examine statistical significance, with a significance threshold of p<0.05.

## RESULTS

### Effects of garlic skin on growth performance in goats

**Figure 1:**
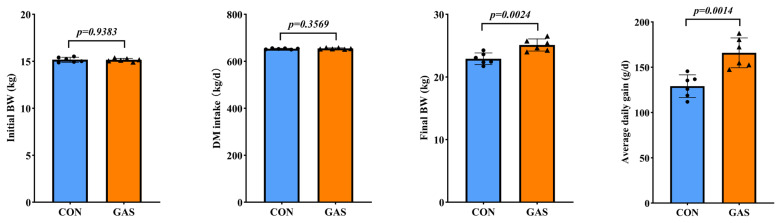
Effects of garlic skin on growth performance in goats. ● represents the data points for the CON group. ▲ represents the data points for the GAS group. CON, control group fed the basal diet; GAS, fed the basal diet supplemented with 16% garlic skin. p<0.05 indicates that there is a significant difference between the two groups.

The addition of garlic skin to the diet improved the growth performance of goats (Figure 1). Compared with the CON group, the GAS group presented significantly greater FW and ADG (p<0.05). However, there was no significant effect on DMI in goats (p>0.05).

### Effects of garlic skin on serum antioxidant capacity in goats

**Figure 2:**
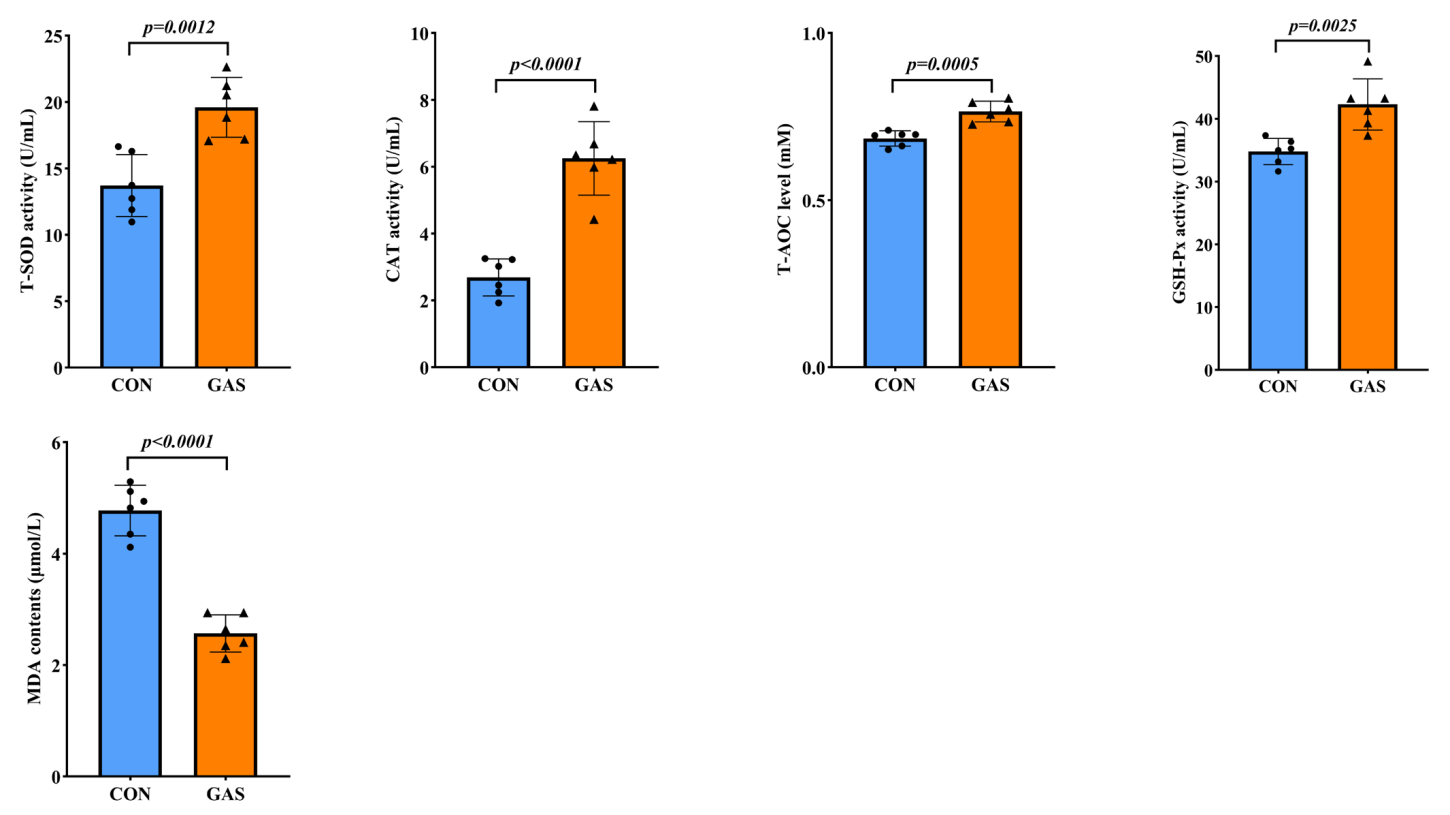
Effects of garlic skin on serum antioxidant capacity in black goats. ● represents the data points for the CON group. ▲ represents the data points for the GAS group. CON, control group fed the basal diet; GAS, fed the basal diet supplemented with 16% garlic skin. p<0.05 indicates that there is a significant difference between the two groups. T-SOD, total superoxide dismutase; CAT, catalase; T-AOC, total antioxidant capacity; GSH-Px, glutathione peroxidase; MDA, malondialdehyde.

The addition of garlic skin to the diet had a positive effect on the serum antioxidant capacity of the goats (Figure 2). Compared with the CON group, the serum T-SOD, GSH-Px, CAT, and T-AOC levels were significantly greater in the GAS group, whereas the MDA contents was significantly lower (p<0.05).

### Effects of garlic skin on serum biochemical indicators in goats

**Figure 3:**
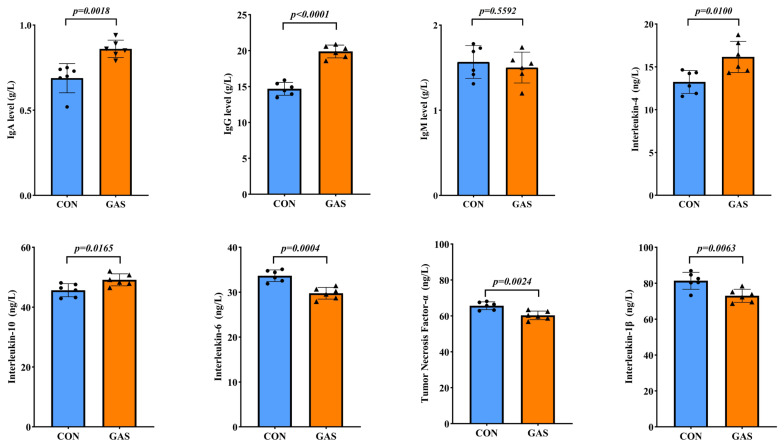
Effects of garlic skin on serum biochemical indicators in goats. ● represents the data points for the CON group. ▲ represents the data points for the GAS group. CON, control group fed the basal diet; GAS, fed the basal diet supplemented with 16% garlic skin. p<0.05 indicates that there is a significant difference between the two groups. IgA, immunoglobulin A; IgG, immunoglobulin G; IgM, immunoglobulin M.

The addition of garlic skin to the diet positively affected the serum immune function of the goats (Figure 3). Compared with the CON group, the GAS group presented significantly higher serum levels of IgA and IgG (p<0.05). Furthermore, the anti-inflammatory cytokines IL-4 and IL-10 were significantly increased, while the proinflammatory cytokines IL-1β, IL-6, and TNF-α were significantly reduced (p<0.05).

### Effects of garlic skin on rumen fermentation parameters in goats

**Figure 4:**
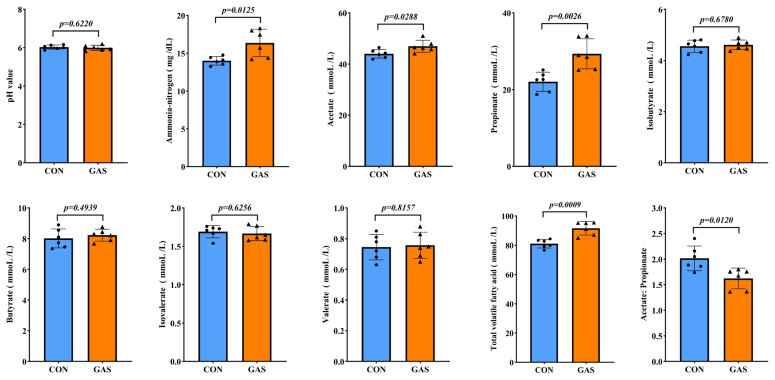
Effects of garlic skin on rumen fermentation parameters in goats. ● represents the data points for the CON group. ▲ represents the data points for the GAS group. CON, control group fed the basal diet; GAS, fed the basal diet supplemented with 16% garlic skin. p<0.05 indicates that there is a significant difference between the two groups.

The inclusion of garlic skin in the diet promoted rumen fermentation parameters and supported rumen health in goats (Figure 4). There was no significant difference in the pH of the rumen fluid between the CON and GAS groups (p>0.05). Compared with the CON group, the GAS group presented significantly greater levels of ammonia-nitrogen, acetate, propionate, and TVFAs in the rumen fluid, whereas the acetate-to-propionate ratio was significantly lower (p<0.05). However, the inclusion of garlic skin in the diet had no significant effect on the levels of isobutyrate, butyrate, isovalerate, or valerate in the rumen fluid (p>0.05).

### Effects of garlic skin on rumen morphology in goats

**Figure 5:**
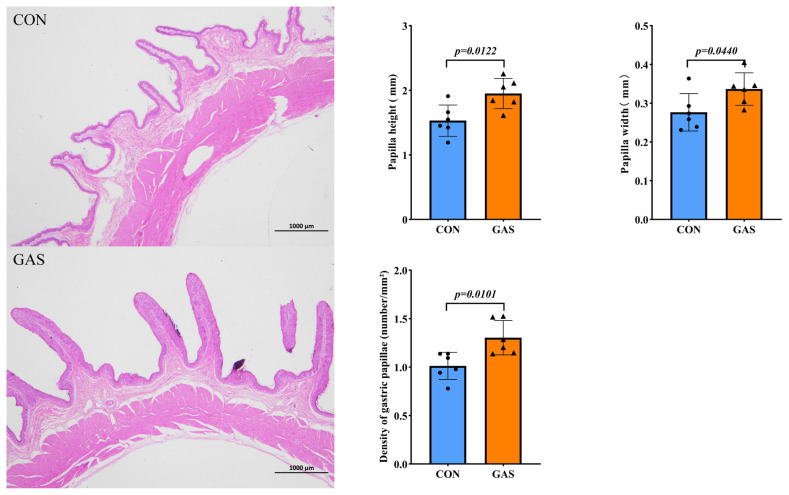
Effects of garlic skin on rumen morphology in goats. ● represents the data points for the CON group. ▲ represents the data points for the GAS group. CON, control group fed the basal diet; GAS, fed the basal diet supplemented with 16% garlic skin. The value between two groups are significant (p<0.05).

The inclusion of garlic skin in the diet improved rumen morphology in goats (Figure 5). Compared with the CON group, the GAS group presented significantly greater papilla height, papilla width, and density of gastric papillae (p<0.05).

### Effects of garlic skin on the rumen microbiota in goats

In the CON group, 1,485 ASVs were found, whereas 2,291 ASVs were found in the GAS group. Of these, 1,050 ASVs were shared by the two groups. According to these results, feeding garlic skin raised the rumen fluid’s ASV count (Supplement 3A). The ACE, Chao1, observed species, and Shannon indices were considerably higher in the GAS group than in the CON group, according to alpha diversity analysis (p<0.05, Supplements 3B–3F). Significant variations in the microbial community structure between the CON and GAS groups were found using PCoA based on weighted UniFrac distance and beta diversity analysis (p = 0.04). PC1 and PC2 explained 31.45% and 21.66% of the variance, respectively, with a clear separation of samples from the two groups (Supplement 3G). Analysis of unweighted and weighted UniFrac distance matrices further revealed significant differences in the microbial community structure between the CON and GAS groups. Differences in intragroup and intergroup distances were evident for both weighted and unweighted distance matrices (Supplements 3H, 3I). These results suggest that feeding garlic skin affects the species composition and relative abundance of the rumen microbiota, consequently influencing microbial community functions.

**Figure 6:**
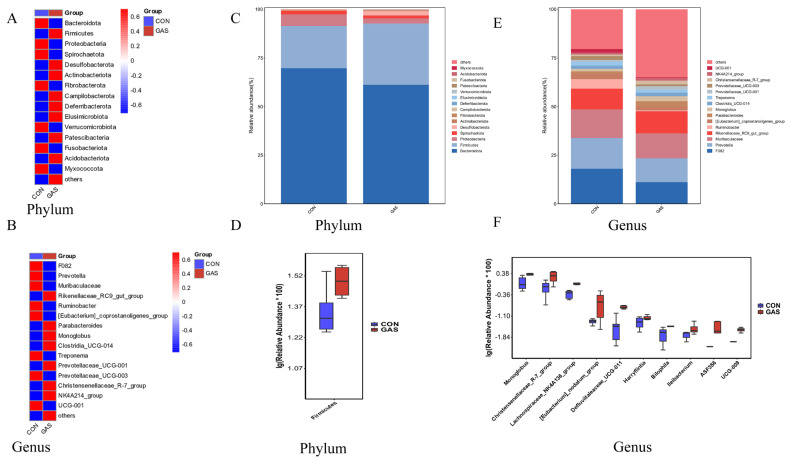
Effects of garlic skin supplementation on rumen microbial diversity and composition in goats. (A) Heatmap of the top 15 bacterial phyla based on relative abundance. (B) Heatmap of the top 15 bacterial genera. (C) Relative abundance of the top 15 bacterial phyla. (D) Boxplots of the top 10 significantly different bacterial phyla between groups (p<0.05). (E) Relative abundance of the top 15 bacterial genera. (F) Boxplots of the top 10 significantly different bacterial genera between groups (p<0.05). CON, control group fed the basal diet; GAS, fed the basal diet supplemented with 16% garlic skin.

Significant changes in the microbial composition between the CON and GAS groups were found when we examined the differences in the microbiota at the phylum and genus levels. This was demonstrated by the different relative abundances of different taxa in the two groups (Figures 6A, 6B). At the phylum level, the dominant phyla were *Bacteroidota* and *Firmicutes*, accounting for more than 90% of the total microbiota. Compared with the CON group, the GAS group presented a significant increase in the relative abundance of *Firmicutes* (17.67% vs. 37.51%, p<0.05; Figures 6C, 6D). At the genus level, the dominant genera included *F082*, *Prevotella*, *Rikenellaceae_RC9_gut_group*, and *Ruminobacter* (Figure 6E). Among the top 10 genera with significant differences, the GAS group presented significant increases in the relative abundances of *Monoglobus*, *Christensenellaceae_R-7_group*, *Harryflintia*, *Lachnospiraceae_NK4A136_group*, *[Eubacterium]_nodatum_group*, *Defluviitaleaceae_UCG-011*, *Ileibacterium*, *ASF356*, and *UCG-009* compared with those of the CON group (p<0.05; Figure 6F).

**Figure 7:**
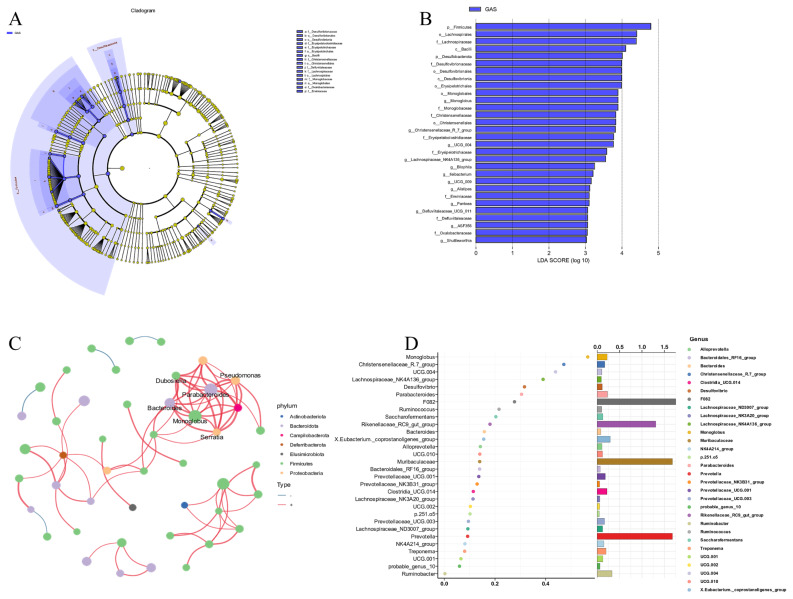
Identification of key microorganisms in goat rumen fluid. (A) Cladogram of annotated differential species. (B) Score plot of differential species based on LDA analysis. (C) Microbial correlation network diagram. (D) Key genera importance plot based on random forest analysis. CON, control group fed the basal diet; GAS, fed the basal diet supplemented with 16% garlic skin.

To explore the effects of garlic skin on specific microbial taxa, LEfSe analysis was performed to reveal differential species between the CON and GAS groups. The two groups’ microbial community structures differed significantly, as seen in Figures 7A, 7B. Using LDA>4.0 as the threshold, we identified *Firmicutes* (phylum), *Lachnospirales* (order), *Lachnospiraceae* (family), and *Bacilli* (class) as potential biomarkers, with their relative abundances significantly increasing in the GAS group, whereas no biomarkers were identified in the CON group. Additionally, a microbial correlation network analysis (Figure 7C) demonstrated that garlic skin supplementation significantly altered the interaction relationships among rumen microorganisms in goats. The results revealed increased positive correlations among specific taxa, promoting microbial community synergy and functional stability. Core taxa, including *Monoglobus*, *Parabacteroides*, *Serratia*, *Bacteroides*, *Dubosiela*, and *Pseudomonas*, were identified as potentially critical players in modulating the rumen ecosystem in response to garlic skin supplementation. Random forest analysis (Figure 7D) further confirmed that garlic skin significantly reshaped the composition and structure of the goat rumen microbiota. Changes in the abundances of *Monoglobus*, *Christensenellaceae_R-7_group*, *Lachnospiraceae_NK4A136_group*, and *Desulfovibrio* suggest that garlic skin may influence rumen metabolic functions by regulating specific microbial genera. These results offer theoretical justification for more research on the possible advantages of garlic skin for goat rumen function.

### Effects of garlic skin on goat rumen microbial metabolomics

**Figure 8:**
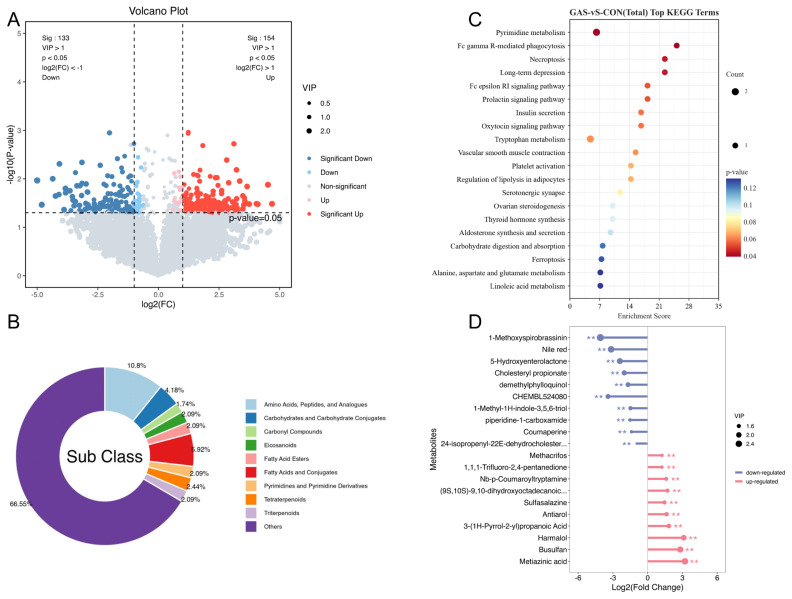
Effects of garlic skin on rumen microbial metabolomics in goats. (A) Volcano plot of differential metabolites. Red dots represent upregulated metabolites in the GAS group, blue dots represent downregulated metabolites, and gray dots represent non-significant metabolites. The x-axis indicates log2 (FC) values, and the y-axis indicates −log10 (p-value). (B) Lollipop chart of the top 10 significantly upregulated and downregulated metabolites with the smallest p-values. The x-axis shows log2 (FC), and the y-axis represents differential metabolites. (C) Classification of differential metabolites. (D) KEGG bubble plot of the top 20 enriched pathways. The x-axis represents the enrichment score, while the y-axis lists the pathway information. Larger bubbles indicate more differential metabolites within the pathway, and the bubble color gradient (blue to red) reflects the p-value, with smaller values indicating higher significance. CON, control group fed the basal diet; GAS, fed the basal diet supplemented with 16% garlic skin. KEGG, Kyoto Encyclopedia of Genes and Genomes.

The rumen microbial metabolome contained 5,915 compounds in total (Supplement 4A). Rumen metabolites in the two groups were easily differentiated using OPLS-DA score plots (Supplement 4B). Using the criterion of VIP>1, p<0.05, and FC>2 or <1/2, differential metabolites were evaluated, and 287 substantially different metabolites between the CON and GAS groups were found. Figure 8A shows that 133 of these metabolites were downregulated and 154 were upregulated. Detailed information on the top 20 significantly upregulated and downregulated metabolites is provided in Supplement 5. To visually present the most significant differential metabolites, the top 10 upregulated and downregulated metabolites with the smallest p values were illustrated via lollipop charts (Figure 8D). Analysis of these 287 differentially abundant metabolites revealed that 10.8% were amino acids, peptides, and analogs; 5.92% were fatty acids and conjugates; 2.44% were tetraterpenoids; and 66.55% were other compounds (Figure 8B). KEGG pathway analysis revealed that these differentially abundant metabolites were enriched primarily in pathways such as pyrimidine metabolism, Fc gamma R-mediated phagocytosis, necroptosis, long-term depression, and the Fc epsilon RI signaling pathway (Figure 8C).

### Combined metagenome and metabolome analysis

**Figure 9:**
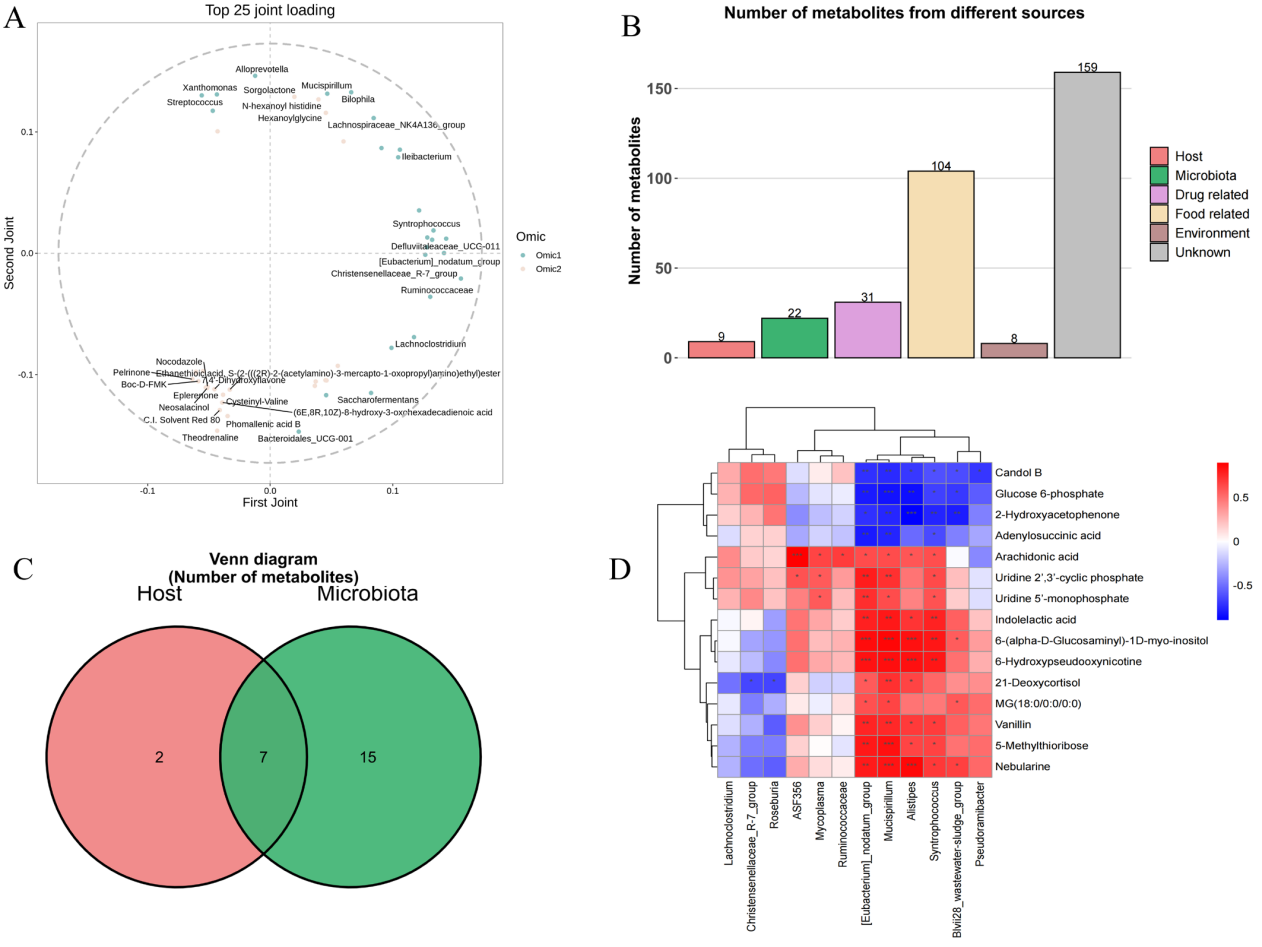
Combined metagenome and metabolome analysis. (A) O2PLS analysis of metagenome and metabolome data to identify key correlations. (B, C) Metabolic pathway enrichment analysis of metabolites using MetOrigin 2.0. (D) Correlation analysis between target metabolites and microorganisms, with green markers indicating negative regulatory correlations. CON, control group fed the basal diet; GAS, fed the basal diet supplemented with 16% garlic skin.

We conducted an integrated analysis of the two datasets to explore potential correlations between the metagenome and metabolome. O2PLS analysis identified the top 25 microbes and metabolites with the strongest association effects (Figure 9A). Further enrichment analysis via MetOrigin 2.0 and metabolic pathway enrichment analysis (MPEA) was performed to filter out host-derived metabolites, retaining 22 metabolites that were either microbially derived or jointly produced by the host and microbes (Figures 9B, 9C). These 22 metabolites were further screened using a fold change (FC) threshold of ≥2.5, resulting in the identification of 15 significant differential metabolites (Supplement 6). Additionally, O2PLS analysis with a significance threshold of p≤0.05 identified seven microbes among the top 25 candidates (Supplement 7). To investigate the relationships between the 15 metabolites and the seven microbes, Pearson correlation analysis was conducted. The results revealed negative regulatory correlations between microbes and metabolites highlighted in green in the correlation heatmap (Figure 9D).

**Figure 10:**
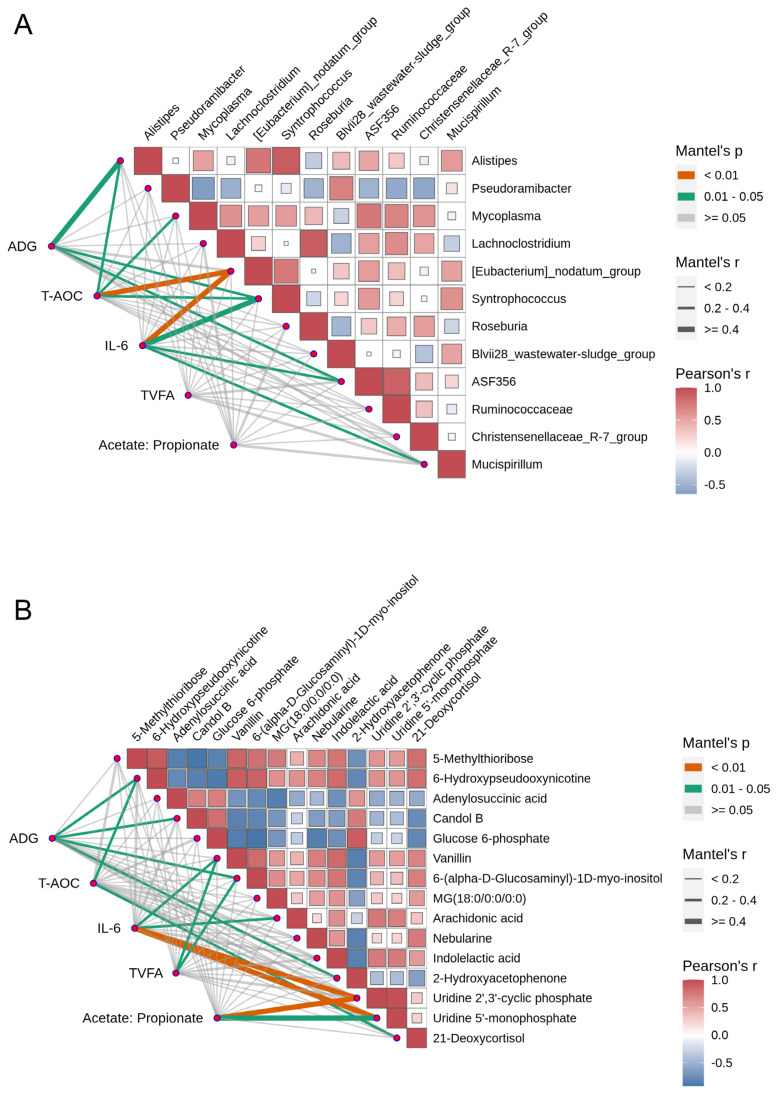
Correlation analysis between microorganisms, metabolites, and production performance. (A) Correlation analysis between microorganisms and production performance. (B) Correlation analysis between metabolites and production performance. ADG, average daily gain; T-AOC, total antioxidant capacity; IL-6, interleukin-6; TVFA, total volatile fatty acids.

To evaluate the impacts of the 12 selected microorganisms and 15 metabolites on goat growth performance, antioxidant capacity, and rumen health, correlation analyses were performed between these microorganisms/metabolites and ADG, T-AOC, IL-6, TVFA, and the acetate-to-propionate ratio. As shown in Figure 10A, different microorganisms had significantly different effects on the various indicators. *Alistipes*, *Pseudoramibacter*, and *Mycoplasma* were significantly positively correlated with T-AOC and IL-6 (r≥0.4, p<0.05), whereas *[Eubacterium]_nodatum_group* was strongly positively correlated with the acetate-to-propionate ratio (r>0.5). Additionally, *Syntrophococcus* and *Roseburia* were significantly positively correlated with multiple indicators, including T-AOC and IL-6. As shown in Figure 10B, different metabolites had significantly different effects on the various indicators. Several metabolites presented significant correlations with ADG, T-AOC, TVFA, the acetate-to-propionate ratio, and IL-6 (|r|≥0.3). 5-Methylthioribose and 6-hydroxypseudooxynicotine were strongly positively correlated with ADG (r>0.4, p<0.01), whereas glucose, 6-phosphate and vanillin were significantly positively correlated with T-AOC (r>0.4, p<0.01). Additionally, vanillin and 6-(alpha-D-glucosamine)-1D-myo-inositol promoted TVFA (r>0.3, p<0.01). These findings suggest that certain metabolites, such as 5-methylthioribos and glucose 6-phosphate, may serve as important potential biomarkers for enhancing growth performance and antioxidant capacity. These results provide valuable insights for further investigations into their biological mechanisms.

## DISCUSSION

Garlic products are rich in bioactive substances that can improve immune function, thereby enhancing animal production performance. They may also serve as alternatives to growth-promoting feed antibiotics [13]. Our study revealed that feeding garlic skin in the diet significantly improved the FW and ADG of goats. The bioactive substances found in garlic skin, particularly polyphenols, improve energy metabolism. Therefore, better energy use may be partly responsible for the higher growth rate seen in the GAS category. Garlic skin is rich in bioactive compounds with demonstrated antibacterial, antiviral, and antioxidant activities, which may synergistically support physiological homeostasis and oxidative balance. Consistent with our findings, feeding garlic skin significantly increased the serum levels of T-SOD, GSH-Px, CAT, and T-AOC in goats while significantly reducing the MDA level [5]. These increases in antioxidant enzyme activities indicate an enhanced antioxidant defense system. In this study, the serum levels of IgA, IgG, IL-4, and IL-10 were significantly increased in the GAS group, while the concentrations of IL-1β, IL-6, and TNF-α were significantly reduced. These findings demonstrate that garlic skin supplementation can enhance immune function and modulate inflammatory responses in goats. These immunomodulatory effects are consistent with previous studies showing that garlic and its derivatives can enhance immune function and reduce inflammatory cytokine expression in ruminants [14,15]. Collectively, our findings suggest that the improved growth performance observed in the GAS group may be partly attributed to enhanced immune status and attenuated inflammation.

Ruminants primarily consume roughage rich in cellulose, which needs to be degraded into VFAs by microbial action before it can be absorbed and utilized. Generally, rumen cellulolytic bacteria exhibit greater activity when the rumen pH ranges from 6.00–6.8 [16]. The primary source of ammonia-nitrogen in the rumen is the metabolism of nitrogen from food, both protein and nonprotein (e.g., urea and amino acids). It provides nitrogen for microbial protein synthesis and serves as an indicator of the fermentation status of the rumen. Garlic skin supplementation increased the concentration of ruminal NH_3_-N, which may reflect enhanced dietary protein degradation. Although elevated NH_3_-N can lead to nitrogen loss via hepatic urea synthesis, the simultaneous increases in VFAs and the abundance of beneficial microbial genera suggest that much of the ammonia may have been effectively utilized for microbial protein synthesis. These findings align with previous studies demonstrating that garlic products promote microbial nitrogen utilization in the rumen [17]. In this study, feeding garlic skin increased the concentrations of acetate, propionate, and TVFAs in goat rumen fluid while reducing the Acetate: Propionate. These findings are consistent with other studies on garlic products in ruminants [9,18]. Rumen papillae are the primary sites for absorption of VFAs and play a vital role in maintaining rumen health and nutrient utilization efficiency in ruminants. Small molecules produced during roughage digestion are absorbed through the rumen wall into the bloodstream, providing essential nutrients for the animal. An increased height of the rumen papillae enhances the absorption surface area for VFAs [19]. Our study demonstrated that feeding garlic skin improved rumen morphology, which was likely associated with increased concentrations of propionate and butyrate in the rumen [20].

The diversity of the rumen microbiota in ruminants significantly affects nutrient digestion, metabolism, and fermentation status, making the rumen a crucial organ with protective, immune, and nutritional functions [21]. Our study, which used 16S rDNA high-throughput sequencing, revealed that feeding garlic skin significantly increased the alpha diversity of the rumen microbiota in goats. This higher microbial diversity contributes to more efficient nutrient breakdown, improved feed utilization, and enhanced growth and health of the animals [22]. At the phylum level, the relative abundance of *Firmicutes* in the GAS group significantly increased. Many bacteria within the *Firmicutes* phylum produce cellulases that degrade cellulose into monosaccharides such as glucose, which are subsequently fermented into VFAs to provide energy for ruminants [23]. These results are consistent with previous studies showing that garlic and its derivatives can increase the abundance of *Firmicutes* in the gastrointestinal tract of animals [24]. At the genus level, the GAS group presented a significant increase in the abundance of *Monoglobus*, *Christensenellaceae_R-7_group*, *Lachnospiraceae_NK4A136_group*, *[Eubacterium]_nodatum_group*, *Defluitaleaceae_UCG-011*, *Harryflintia*, *Bilophila*, *Ileibacterium*, *ASF356*, and *UCG-009.* Among these, *Monoglobus* plays a role in the breakdown and metabolism of carbohydrates and polysaccharides in the rumen, converting them into VFAs that serve as an energy source for ruminants [25]. *Christensenellaceae_R-7_group*, *Harryflintia*, and *Lachnospiraceae_NK4A136_group* contribute to carbohydrate metabolism, cellulose degradation, VFA production, and nitrogen utilization, which promote protein synthesis [26]. Moreover, *Defluviitaleaceae_UCG-011*, *Ileibacterium*, *ASF356*, and *UCG-009* play roles in breaking down nutrients in feed and providing energy and nutrients to the host, either through direct activity or through interactions with other microbes [27]. Additionally, *Firmicutes* (phylum), *Lachnospirales* (order), *Lachnospiraceae* (family), and *Bacilli* (class) were identified as potential biomarkers in the GAS group. These microorganisms are crucial for cellulose degradation, carbohydrate fermentation, protein and nitrogen metabolism, immune enhancement, and maintaining the microecological balance of the rumen. Together, these compounds play a vital role in sustaining rumen health [28]. These results suggest that garlic skin not only promotes beneficial microbial genera, but also functionally resembles antibiotic feed additives in shaping rumen microbial ecology. Notably, the increase in *Firmicutes* and fiber-degrading genera such as *Monoglobus* and *Christensenellaceae_R-7_group* indicates enhanced fiber fermentation and energy metabolism. Prior studies have shown that garlic-based additives can exert antimicrobial effects similar to monensin while avoiding adverse side effects, supporting their use as natural antibiotic alternatives in ruminant production systems [5].

Metabolites such as methacrifos, (9S,10S)-9,10-dihydroxyoctadecanoic acid, sulfasalazine, and harmalol were upregulated in the rumens of the GAS group. These compounds are known to reduce oxidative stress and inflammation, enhance host immune function, promote rumen microbial balance, and improve antimicrobial capacity [29]. Conversely, 1-methyl-1H-indole-3,5,6-triol, an indole derivative, was downregulated in the GAS group, indicating that feeding garlic skin reduces the risk of inflammation and improves the rumen environment [30]. A total of 12 microbial taxa and 15 metabolites were identified as potentially playing key roles in improving goat production performance following garlic skin supplementation. Regulatory relationships between these metabolites and microorganisms were also observed. The increases in *Alistipes*, *Pseudoramibacter*, and *Mycoplasma* were significantly positively correlated with T-AOC and IL-6, suggesting that these microbial populations may increase antioxidant levels while moderately stimulating immune responses [31]. The increase in *Syntrophococcus* and *Roseburia* further strengthened antioxidant and immune regulatory functions. Specifically, *Roseburia*, a known butyrate-producing bacterium, likely contributes to improving gut barrier function and indirectly supports overall health [32]. Garlic skin modulated the rumen microbial community structure by increasing the proportion of beneficial microbes, increasing metabolic efficiency, and suppressing harmful bacteria. This resulted in improved antioxidant capacity, reduced inflammation, and optimized energy metabolism. These findings demonstrate the potential of garlic skin as a feed additive to improve the rumen microbiota, enhance animal health, and increase production performance.

Furthermore, we found that 5-methylthioribose was positively correlated with ADG following garlic skin supplementation. As an intermediate product in the methionine cycle, 5-methylthioribose may increase nitrogen metabolism and protein synthesis in rumen microorganisms, thereby improving nutrient utilization efficiency and promoting growth performance [33]. Additionally, glucose 6-phosphate and vanillin were positively correlated with T-AOC, indicating their potential roles in enhancing antioxidant enzyme activity or directly scavenging free radicals to improve antioxidant capacity. Vanillin was also positively correlated with TVFA, suggesting that it may optimize rumen microbial fermentation processes to further increase energy metabolism efficiency. These findings reveal the mechanisms by which garlic skin links growth performance, antioxidant capacity, and energy metabolism through regulating metabolites such as 5-methylthioribose, glucose-6-phosphate, and vanillin.

## CONCLUSION

This study highlights the potential of garlic skin as a feed ingredient to enhance growth performance, antioxidant capacity, and immune function in goats. Garlic skin improved nutrient utilization by modulating rumen fermentation, increasing VFA production, and optimizing the acetate-to-propionate ratio. It also increased antioxidant enzyme activities, reduced oxidative stress, and regulated cytokine levels, improving overall immune and metabolic health. Rumen microbiome analysis revealed increased microbial diversity and enrichment of beneficial taxa, such as *Firmicutes* and *Christensenellaceae_R-7_group*, which promoted fiber degradation and VFA production. Key metabolites, such as 5-methylthioribose and glucose 6-phosphate, were positively associated with growth and antioxidant capacity. In conclusion, garlic skin supplementation is a sustainable strategy to improve ruminant health and productivity, offering valuable insights for the efficient utilization of this agro-industrial byproduct.

## SUPPLEMENTARY MATERIAL

**Supplement 1.** Nutritional composition and bioactive compounds of garlic skin.

**Supplement 2.** Diet formulation and nutritional composition.

**Supplement 3.** Effects of garlic skin on rumen fluid microbiota in goats.

**Supplement 4.** Effects of garlic skinl on the rumen microbiome metabolomics of goats.

**Supplement 5.** Top 20 Significantly Upregulated and Downregulated Metabolites.

**Supplement 6.** Fifteen differential metabolites screened based on the analysis of MetOrigin 2.0.

**Supplement 7.** Fifteen differential metabolites screened based on O2PLS analysis.

## References

[1] Lee YH Kim YI Oh YK Ahmadi F Kwak WS Yield survey and nutritional evaluation of garlic stalk for ruminant feed J Anim Sci Technol 2017 59 22 10.1186/s40781-017-0147-3 29085659 PMC5651561

[2] Ma T Chen D Tu Y Effect of supplementation of allicin on methanogenesis and ruminal microbial flora in Dorper crossbred ewes J Anim Sci Biotechnol 2016 7 1 10.1186/s40104-015-0057-5 26779340 PMC4714447

[3] Imbabi T Hassan TMM Osman A Impacts of thyme and/or garlic oils on growth, immunity, antioxidant and net farm income in Damascus goats Sci Rep 2024 14 13173 10.1038/s41598-024-62417-0 38849384 PMC11161640

[4] Liu TW Pang R Huang L Effects of allicin addition on growth performance, rumen microbiome, and ruminal epithelial proteome of high-grain-fed goats Anim Feed Sci Technol 2024 310 115944 10.1016/j.anifeedsci.2024.115944

[5] Tang Y Huang L Sun X Effects of allicin on growth performance, antioxidant profile, and microbiota compared to monensin of growing goats Anim Sci J 2024 95 e13917 10.1111/asj.13917 38323750

[6] Qureshi AA Din ZZ Abuirmeileh N Suppression of avian hepatic lipid metabolism by solvent extracts of garlic: impact on serum lipids J Nutr 1983 113 1746 55 10.1093/jn/113.9.1746 6886822

[7] Ichikawa M Ryu K Yoshida J Identification of six phenylpropanoids from garlic skin as major antioxidants J Agric Food Chem 2003 51 7313 7 10.1021/jf034791a 14640577

[8] Xu Y Yi M Sun S The regulatory mechanism of garlic skin improving the growth performance of fattening sheep through metabolism and immunity Front Vet Sci 2024 11 1409518 10.3389/fvets.2024.1409518 38872796 PMC11171129

[9] Zhu W Su Z Xu W Garlic skin induces shifts in the rumen microbiome and metabolome of fattening lambs Animal 2021 15 100216 10.1016/j.animal.2021.100216 34051409

[10] Zhou X Shen X Environmental sulfur and molybdenum stress disrupts mineral homeostasis and induces physiological and molecular alterations in goats Environ Res 2025 282 122033 10.1016/j.envres.2025.122033 40451414

[11] Zhou X Shen X Multi-omics insights into the mechanisms of muscle damage induced by molybdenum exposure in goats Ecotoxicol Environ Saf 2025 299 118373 10.1016/j.ecoenv.2025.118373 40413927

[12] Ren H Shen X Multi-omics reveals the hepatic metabolic mechanism of neurological symptoms caused by selenium exposure in Przewalski’s gazelle (Procapra przewalskii) Environ Pollut 2025 375 126341 10.1016/j.envpol.2025.126341 40316242

[13] Ding H Ao C Zhang X Potential use of garlic products in ruminant feeding: a review Anim Nutr 2023 14 343 55 10.1016/j.aninu.2023.04.011 37635929 PMC10448032

[14] Hitchcock JK Mkwanazi N Barnett C The garlic compound Z-Ajoene, S-Thiolates COX2 and STAT3 and dampens the inflammatory response in RAW264.7 macrophages Mol Nutr Food Res 2021 65 2000854 10.1002/mnfr.202000854 33274836

[15] Wu J Yu G Zhang X A fructan-type garlic polysaccharide upregulates immune responses in macrophage cells and in immunosuppressive mice Carbohydr Polym 2024 344 122530 10.1016/j.carbpol.2024.122530 39218552

[16] Viennasay B Wanapat M Strategic supplementation of Flemingia silage to enhance rumen fermentation efficiency, microbial protein synthesis and methane mitigation in beef cattle BMC Vet Res 2020 16 480 10.1186/s12917-020-02703-x 33298016 PMC7726859

[17] Zhu Z Mao S Zhu W Effects of ruminal infusion of garlic oil on fermentation dynamics, Fatty Acid profile and abundance of bacteria involved in biohydrogenation in rumen of goats Asian-Australas J Anim Sci 2012 25 962 70 10.5713/ajas.2011.11442 25049651 PMC4092973

[18] Patra AK Kamra DN Agarwal N Effects of extracts of spices on rumen methanogenesis, enzyme activities and fermentation of feeds in vitro J Sci Food Agric 2010 90 511 20 10.1002/jsfa.3849 20355074

[19] Lesmeister KE Tozer PR Heinrichs AJ Development and analysis of a rumen tissue sampling procedure J Dairy Sci 2004 87 1336 44 10.3168/jds.S0022-0302(04)73283-X 15290981

[20] Beiranvand H Ghorbani GR Khorvash M Interactions of alfalfa hay and sodium propionate on dairy calf performance and rumen development J Dairy Sci 2014 97 2270 80 10.3168/jds.2012-6332 24508441

[21] Bickhart DM Weimer PJ Symposium review: host–rumen microbe interactions may be leveraged to improve the productivity of dairy cows J Dairy Sci 2018 101 7680 9 10.3168/jds.2017-13328 29102146

[22] Amin AB Mao S Influence of yeast on rumen fermentation, growth performance and quality of products in ruminants: a review Anim Nutrf 2021 7 31 41 10.1016/j.aninu.2020.10.005 PMC8110857 33997329

[23] Gharechahi J Vahidi MF Sharifi G Lignocellulose degradation by rumen bacterial communities: new insights from metagenome analyses Environ Res 2023 229 115925 10.1016/j.envres.2023.115925 37086884

[24] Ha J Kim J Kim S Lee KJ Shin H Garlic-Induced enhancement of bifidobacterium: enterotype-specific modulation of gut microbiota and probiotic populations Microorganisms 2024 12 1971 10.3390/microorganisms12101971 39458280 PMC11509698

[25] Shao P Sha Y Liu X Supplementation with astragalus root powder promotes rumen microbiota density and metabolome interactions in lambs Animals 2024 14 788 10.3390/ani14050788 38473173 PMC10931105

[26] Yu S Li L Zhao H Characterization of the dynamic changes of ruminal microbiota colonizing citrus pomace waste during rumen incubation for volatile fatty acid production Microbiol Spectr 2023 11 e03517 22 10.1128/spectrum.03517-22 36862010 PMC10101060

[27] Cui G Li S Ye H Gut microbiome and frailty: insight from genetic correlation and mendelian randomization Gut Microbes 2023 15 2282795 10.1080/19490976.2023.2282795 37990415 PMC10730212

[28] Cao Y Feng T Wu Y The multi-kingdom microbiome of the goat gastrointestinal tract Microbiome 2023 11 219 10.1186/s40168-023-01651-6 37779211 PMC10544373

[29] Olmez D Babayigit A Uzuner N Efficacy of sulphasalazine on lung histopathology in a murine model of chronic asthma Exp Lung Res 2008 34 501 11 10.1080/01902140802271859 18850376

[30] Xu W Gavia DJ Tang Y Biosynthesis of fungal indole alkaloids Nat Prod Rep 2014 31 1474 87 10.1039/C4NP00073K 25180619 PMC4162825

[31] Wu L Xie X Li Y Gut microbiota as an antioxidant system in centenarians associated with high antioxidant activities of gut-resident Lactobacillus NPJ Biofilms Microbiomes 2022 8 102 10.1038/s41522-022-00366-0 36564415 PMC9789086

[32] Zhuang Y Liu S Gao D The Bifidobacterium-dominated fecal microbiome in dairy calves shapes the characteristic growth phenotype of host NPJ Biofilms Microbiomes 2024 10 59 10.1038/s41522-024-00534-4 39034349 PMC11271470

[33] Albers E Metabolic characteristics and importance of the universal methionine salvage pathway recycling methionine from 5′-methylthioadenosine IUBMB Life 2009 61 1132 42 10.1002/iub.278 19946895

