# Supplementary material for: Evaluation of garlic skin as a forage source for goats: effects on performance, antioxidant capacity, immune function and ruminal health
Source: Anim Biosci. 2025 Jul 11;39(1):250169. doi: 10.5713/ab.25.0169 (PMC12754484; doi:10.5713/ab.25.0169)
Supplement: Supplementary file 1 [file ab-25-0169-Supplementary-1.pdf]

Supplement 1. Nutritional composition and bioactive compounds of garlic skin

Chemical composition, % DM	Contents
Dry matter	90.85
Crude protein	13.55
Ether extracts	4.32
Ash	5.87
Neutral detergent fibre	42.36
Acid detergent fibre	36.57
Bioactive compounds, mg/g	
Total polyphenols content	6.35
Total flavonoid content	0.48
