# Supplementary material for: Evaluation of garlic skin as a forage source for goats: effects on performance, antioxidant capacity, immune function and ruminal health
Source: Anim Biosci. 2025 Jul 11;39(1):250169. doi: 10.5713/ab.25.0169 (PMC12754484; doi:10.5713/ab.25.0169)
Supplement: Supplementary file 2 [file ab-25-0169-Supplementary-2.pdf]

Supplement 2. Diet formulation and nutritional composition

Ingredients	CON	GAS
Corn straw	25	8
Peanut straw	32	32
Garlic skin	-	16
Soybean meal	15	15
Corn	24	24
Sodium chloride	0.5	0.5
Limestone	0.4	1
Dicalcium phosphate	0.7	0.7
Urea phosphate	0.4	0.8
Premix[1]	2	2
Total	100	100
Nutrient levels[2]		
Dry matter (fresh)	33.25	33.31
Gross energy (MJ/kg)	15.12	15.20
Metabolizable energy (MJ/kg)	9.16	9.15
Crude protein (%)	14.26	14.30
Neutral detergent fiber (%)	42.62	42.69
Acid detergent fiber (%)	21.20	21.22
Calcium (%)	0.94	0.95
Total phosphorus (%)	0.55	0.56

[1]The premix provides the following per kilogram of feed: Vitamin A (VA) 60,000 IU, Vitamin D3 (VD) 39,000 IU, Vitamin E (VE) 75 IU, Niacin 150 mg, Pantothenic acid 45 mg, Biotin 3.0 mg, Copper (Cu) 10 mg, Zinc (Zn) 50 mg, Iron (Fe) 70 mg, Selenium (Se) 0.30 mg, Iodine (I) 5.25 mg, Manganese (Mn) 50 mg, Cobalt (Co) 2.25 mg.

[2]ME was a calculated value，while the others were measured values.
