# Supplementary material for: Evaluation of garlic skin as a forage source for goats: effects on performance, antioxidant capacity, immune function and ruminal health
Source: Anim Biosci. 2025 Jul 11;39(1):250169. doi: 10.5713/ab.25.0169 (PMC12754484; doi:10.5713/ab.25.0169)
Supplement: Supplementary file 3 [file ab-25-0169-Supplementary-3.pdf]

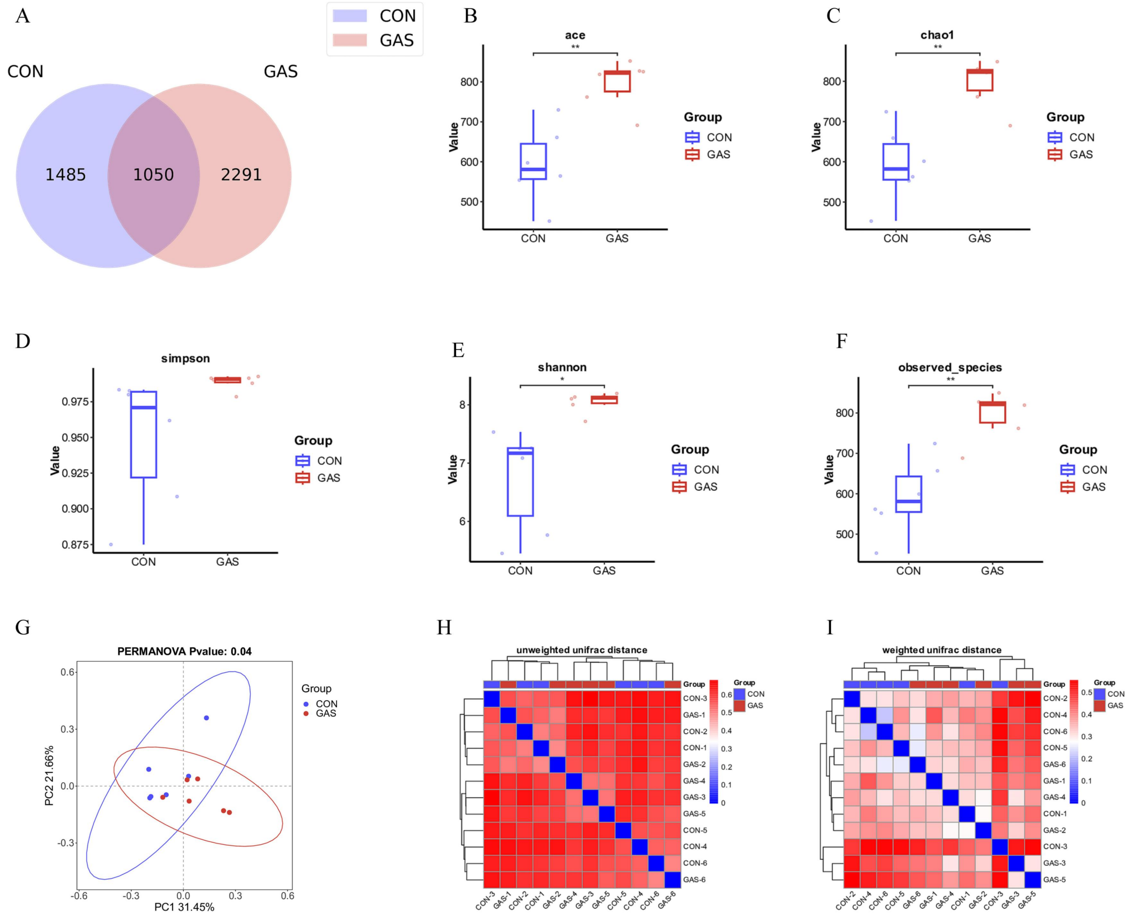

Supplement 3. Effects of garlic skin on rumen fluid microbiota in goats. A, Venn diagram of ASV distribution. B, ACE index. C, Chao1 index. D: Observed species index. E, Shannon index. F, Simpson index. G: Principal coordinates analysis (PCoA). H and I, Heatmaps of sample distances. * indicates a significant difference between the two groups (*$p < 0.05$, **$p < 0.01$). CON, control group fed the basal diet; GAS, fed the basal diet supplemented with 16% garlic skin.
