# Supplementary material for: Evaluation of garlic skin as a forage source for goats: effects on performance, antioxidant capacity, immune function and ruminal health
Source: Anim Biosci. 2025 Jul 11;39(1):250169. doi: 10.5713/ab.25.0169 (PMC12754484; doi:10.5713/ab.25.0169)
Supplement: Supplementary file 4 [file ab-25-0169-Supplementary-4.pdf]

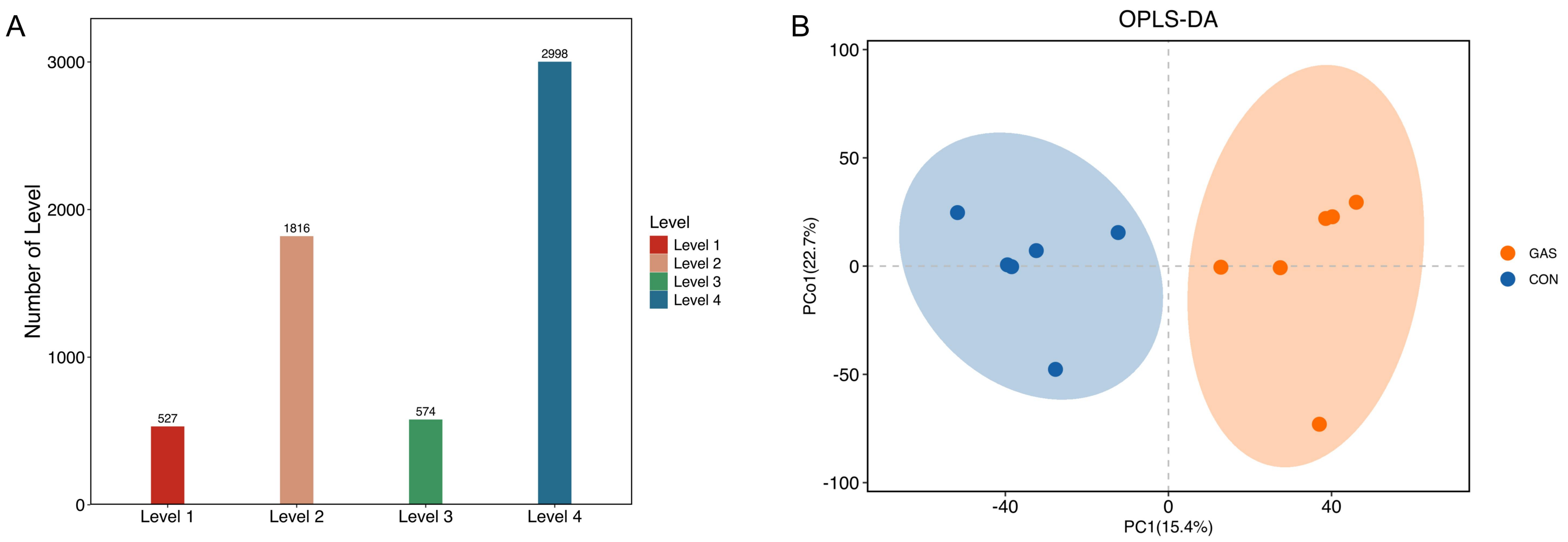

Supplement 4. Effects of garlic skinl on the rumen microbiome metabolomics of goats. A, In rumen microbial metabolites. B, Orthogonal partial least squares discriminant analysis (OPLS-DA) score plot. CON, control group fed the basal diet; GAS, fed the basal diet supplemented with 16%garlic skin.
