# Supplementary material for: Evaluation of garlic skin as a forage source for goats: effects on performance, antioxidant capacity, immune function and ruminal health
Source: Anim Biosci. 2025 Jul 11;39(1):250169. doi: 10.5713/ab.25.0169 (PMC12754484; doi:10.5713/ab.25.0169)
Supplement: Supplementary file 5 [file ab-25-0169-Supplementary-5.pdf]

Supplement 5. Top 20 Significantly Upregulated and Downregulated Metabolites

Metabolites	FoldChange	VIP	p-value	Regulation
Methacrifos	2.3511431	1.4552271	0.0010953	Up
1,1,1-Trifluoro-2,4-pentanedione	2.3397117	1.460036	0.0011554	Up
Harmalol	8.6430411	2.0891159	0.0018993	Up
3-(1H-Pyrrol-2-yl)propanoic Acid	3.5544314	1.7636921	0.002062	Up
Busulfan	7.0337031	2.3158804	0.0041003	Up
Metiazinic acid	9.3127135	2.434303	0.006531	Up
(9S,10S)-9,10-dihydroxyoctadecanoic acid	3.28255	1.6050725	0.0066175	Up
Sulfasalazine	2.7293345	1.6159147	0.0081225	Up
Nb-p-Coumaroyltryptamine	3.0266644	1.5762239	0.0083911	Up
Antiarol	3.106909	1.6705491	0.0087147	Up
N2-Maltulosylarginine	4.3914567	1.5248057	0.0089109	Up
Cocaine-alcohol	5.34531	1.9881413	0.0094626	Up
5-O-Methylleridol	5.1014084	2.1896942	0.0094893	Up
2'-Deoxy-5'-O-thiophosphonouridine	10.209335	2.5288578	0.011148	Up
2-(5-Methyl-2-furanyl)-3-piperidinol	3.2446601	1.7364254	0.0112085	Up
CHEBI:168404	7.4453411	2.3602141	0.0120727	Up
Plaunotol M-6	2.0945765	1.1670566	0.0130988	Up
Hydroquinone sulfate	22.874729	2.9435021	0.0132588	Up
Arachidonic acid	5.4840921	1.8421981	0.0136612	Up
TG(10:0/8:0/8:0)	12.607972	2.5392814	0.0144143	Up
Cholesteryl propionate	0.24674	1.90524	0.00113	Down
24-isopropenyl-22E-dehydrocholesterol	0.49667	1.26919	0.00189	Down
Coumaperine	0.38064	1.44321	0.00342	Down
demethylphylloquinol	0.3094	1.8268	0.00385	Down

Nile red	0.1123	2.45318	0.00458	Down
1-Methoxyspirobrassinin	0.05898	2.6776	0.00493	Down
5-Hydroxyenterolactone	0.18925	2.20534	0.00527	Down
piperidine-1-carboxamide	0.3479	1.4825	0.00593	Down
1-Methyl-1H-indole-3,5,6-triol	0.35731	1.63506	0.00721	Down
CHEMBL524080	0.09329	1.8257	0.00745	Down
Apocynin A	0.44707	1.50893	0.00865	Down
NILOTICIN	0.23424	1.91293	0.00866	Down
Myristicin	0.45531	1.44558	0.00873	Down
9-Tricosene	0.26475	1.63987	0.009	Down
Diffutidin	0.42546	1.28372	0.00927	Down
Eplerenone	0.04871	2.63809	0.00998	Down
Rutacultin	0.02435	3.31809	0.01082	Down
5Z-Pentacosene	0.46271	1.15409	0.01122	Down
Tariquidar	0.11255	2.19892	0.01241	Down
3,4,3',4'-Tetrahydroxypirardixanthin	0.12645	2.39055	0.01278	Down
