# Supplementary material for: Evaluation of garlic skin as a forage source for goats: effects on performance, antioxidant capacity, immune function and ruminal health
Source: Anim Biosci. 2025 Jul 11;39(1):250169. doi: 10.5713/ab.25.0169 (PMC12754484; doi:10.5713/ab.25.0169)
Supplement: Supplementary file 6 [file ab-25-0169-Supplementary-6.pdf]

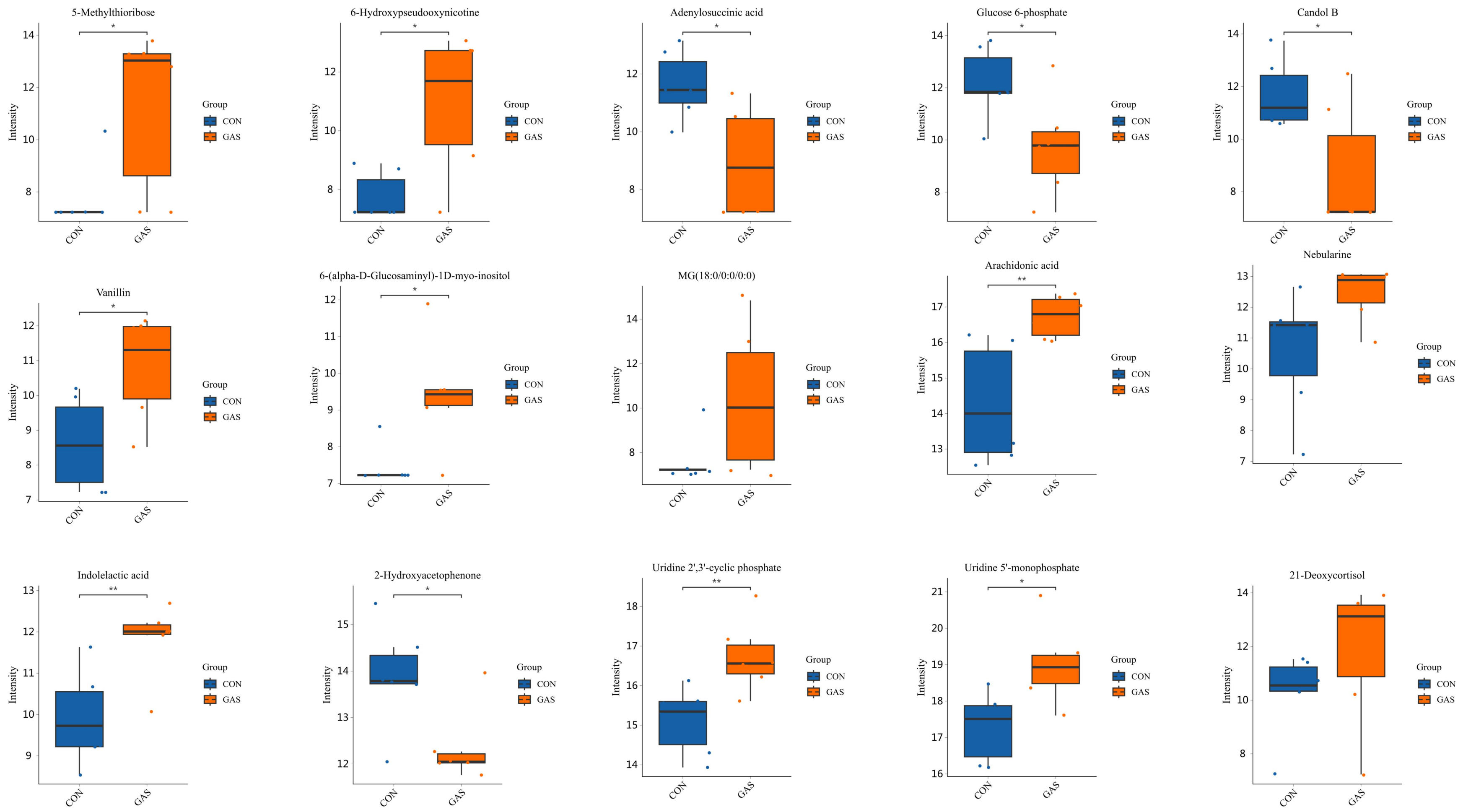

Supplement 6. Fifteen differential metabolites screened based on the analysis of MetOrigin 2.0. CON, control group fed the basal diet; GAS, fed the basal diet supplemented with 16% garlic skin.
